# Supplementary material for: The impact of radiofrequency exposure on Aedes aegypti (Diptera: Culicidae) development
Source: PLoS One. 2024 Feb 27;19(2):e0298738. doi: 10.1371/journal.pone.0298738 (PMC10898727; doi:10.1371/journal.pone.0298738)

PLOS ONE

Impact of Varying Radiofrequency Exposure on Aedes aegypti (Diptera: Culicidae) Development

--Manuscript Draft--

**Manuscript Number:**	PONE-D-23-36469
**Article Type:**	Research Article
**Full Title:**	Impact of Varying Radiofrequency Exposure on Aedes aegypti (Diptera: Culicidae) Development
**Short Title:**	Impact of Varying Radiofrequency Exposure on Aedes aegypti (Diptera: Culicidae) Development
**Corresponding Author:**	Rahmat Dapari<br>Universiti Putra Malaysia<br>Serdang, MALAYSIA
**Keywords:**	Aedes, larval development, radiation, radiofrequency, RF
**Abstract:**	Introduction<br>Wireless communication, connecting billions worldwide, relies on radiofrequency electromagnetic fields (RF-EMF). The advent of fifth-generation (5G) networks shifts RF carriers to higher frequencies. While radio, cell phones, and television have served human needs for decades, higher carrier frequencies pose potential health risks. Insects closely associated with humans, like mosquitoes, may experience increased RF absorption and dielectric heating. This could inadvertently impact their behavior, morphology, and physiology, possibly influencing their spread. This study investigates RF exposure effects on Aedes aegypti mosquitoes, prevalent in indoor environments with higher RF exposure risk. Our focus is on how RF exposure influences egg morphology and Ae. aegypti development.<br>Methods<br>In the experiment, triplicates of 30 eggs were exposed to RF radiation at baseline, 900 MHz, and 18 GHz. Daily observations in an insectarium tracked hatching responses, development times, larval numbers, and pupation periods until adult emergence.<br>Results<br>Results reveal RF radiation affecting the duration of mosquito growth from egg hatching to adult emergence and, although no significant differences in exposure levels were observed, some changes in egg morphology were noted.<br>Conclusion<br>This work highlights the sensitivity of Ae. aegypti egg morphology and development during the aquatic phase to RF radiation, potentially altering their life cycle.
**Order of Authors:**	Rahmat Dapari
	Nik Muhammad Hanif Nik Abdull Halim
	Alya Farzana Mohd Jamili
	Nazri Che Dom
	Nurul Huda Abd Rahman
	Zana Jamal Kareem
**Additional Information:**	
**Question**	**Response**
**Financial Disclosure**<br><br>Enter a financial disclosure statement that describes the sources of funding for the work included in this submission. Review the submission guidelines for detailed requirements. View published research	The author(s) received no specific funding for this work.

Powered by Editorial Manager® and ProduXion Manager® from Aries Systems Corporation

articles from *PLOS ONE* for specific examples.<br><br>This statement is required for submission and **will appear in the published article** if the submission is accepted. Please make sure it is accurate.<br><br>**Funded studies**<br>Enter a statement with the following details:<br>• Initials of the authors who received each award<br>• Grant numbers awarded to each author<br>• The full name of each funder<br>• URL of each funder website<br>• Did the sponsors or funders play any role in the study design, data collection and analysis, decision to publish, or preparation of the manuscript?<br><br>Did you receive funding for this work?	
**Competing Interests**<br><br>Use the instructions below to enter a competing interest statement for this submission. On behalf of all authors, disclose any competing interests that could be perceived to bias this work—acknowledging all financial support and any other relevant financial or non-financial competing interests.<br><br>This statement is required for submission and **will appear in the published article** if the submission is accepted. Please make sure it is accurate and that any funding sources listed in your Funding Information later in the submission form are also declared in your Financial Disclosure statement.<br><br>View published research articles from *PLOS ONE* for specific examples.	The authors have declared that no competing interests exist.

**NO authors have competing interests**<br><br>Enter: *The authors have declared that no competing interests exist.*<br><br>**Authors with competing interests**<br><br>Enter competing interest details beginning with this statement:<br><br>*I have read the journal's policy and the authors of this manuscript have the following competing interests: [insert competing interests here]*<br><br>* typeset	
**Ethics Statement**<br><br>Enter an ethics statement for this submission. This statement is required if the study involved:<br><br>• Human participants<br>• Human specimens or tissue<br>• Vertebrate animals or cephalopods<br>• Vertebrate embryos or tissues<br>• Field research<br><br>Write "N/A" if the submission does not require an ethics statement.<br><br>General guidance is provided below. Consult the submission guidelines for detailed instructions. **Make sure that all information entered here is included in the Methods section of the manuscript.**	The study was approved by the Committee on Animal Research and Ethics (UiTM CARE) under the reference number UiTM CARE: 377/2022(13th May 2022). The approval duration was July 2022 until June 2023.

**Format for specific study types**<br><br>**Human Subject Research (involving human participants and/or tissue)**<br>• Give the name of the institutional review board or ethics committee that approved the study<br>• Include the approval number and/or a statement indicating approval of this research<br>• Indicate the form of consent obtained (written/oral) or the reason that consent was not obtained (e.g. the data were analyzed anonymously)<br><br>**Animal Research (involving vertebrate animals, embryos or tissues)**<br>• Provide the name of the Institutional Animal Care and Use Committee (IACUC) or other relevant ethics board that reviewed the study protocol, and indicate whether they approved this research or granted a formal waiver of ethical approval<br>• Include an approval number if one was obtained<br>• If the study involved *non-human primates*, add *additional details* about animal welfare and steps taken to ameliorate suffering<br>• If anesthesia, euthanasia, or any kind of animal sacrifice is part of the study, include briefly which substances and/or methods were applied<br><br>**Field Research**<br><br>Include the following details if this study involves the collection of plant, animal, or other materials from a natural setting:<br>• Field permit number<br>• Name of the institution or relevant body that granted permission	
**Data Availability**<br><br>Authors are required to make all data underlying the findings described fully available, without restriction, and from the time of publication. PLOS allows rare exceptions to address legal and ethical concerns. See the PLOS Data Policy and FAQ for detailed information.	Yes - all data are fully available without restriction

Powered by Editorial Manager® and ProduXion Manager® from Aries Systems Corporation

A Data Availability Statement describing where the data can be found is required at submission. Your answers to this question constitute the Data Availability Statement and **will be published in the article**, if accepted.<br><br>**Important:** Stating 'data available on request from the author' is not sufficient. If your data are only available upon request, select 'No' for the first question and explain your exceptional situation in the text box.<br><br>Do the authors confirm that all data underlying the findings described in their manuscript are fully available without restriction?	
**Describe where the data may be found in full sentences. If you are copying our sample text, replace any instances of XXX with the appropriate details.**<br><br>• If the data are **held or will be held in a public repository**, include URLs, accession numbers or DOIs. If this information will only be available after acceptance, indicate this by ticking the box below. For example: *All XXX files are available from the XXX database (accession number(s) XXX, XXX.).*<br>• If the data are all contained **within the manuscript and/or Supporting Information files**, enter the following: *All relevant data are within the manuscript and its Supporting Information files.*<br>• If neither of these applies but you are able to provide **details of access elsewhere**, with or without limitations, please do so. For example:<br><br>*Data cannot be shared publicly because of [XXX]. Data are available from the XXX Institutional Data Access / Ethics Committee (contact via XXX) for researchers who meet the criteria for access to confidential data.*<br><br>*The data underlying the results presented in the study are available from (include the name of the third party*	All relevant data are within the manuscript and its Supporting Information files.

and contact information or URL).<br>• This text is appropriate if the data are owned by a third party and authors do not have permission to share the data.<br><br>* typeset	
Additional data availability information:	

Manuscript Click here to access/download;Manuscript;Manuscript.docx

Impact of Varying Radiofrequency Exposure on *Aedes aegypti* (Diptera: Culicidae) Development

Nik Muhammad Hanif Nik Abdull Halim[1,4], Alya Farzana Mohd Jamili[1], Nazri Che Dom[1,2,3], Nurul Huda Abd Rahman[5], Zana Jamal Kareem[6,7], Rahmat Dapari[8*]

[1] Centre of Environmental Health & Safety, Faculty of Health Sciences, Universiti Teknologi MARA (UiTM), UITM Cawangan Selangor, 42300 Puncak Alam, Selangor, Malaysia,

[2] Integrated Mosquito Research Group (I-MeRGe), Universiti Teknologi MARA (UiTM), UITM Cawangan Selangor, 42300 Puncak Alam, Selangor, Malaysia,

[3] Institute for Biodiversity and Sustainable Development (IBSD), Universiti Teknologi MARA, Shah Alam, Malaysia,

[4] Setiu District Health Office, 22100 Bandar Permaisuri, Setiu, Terengganu, Malaysia,

[5] Antenna Research Centre, School of Electrical Engineering, College of Engineering, Universiti Teknologi MARA, 40450 Shah Alam, Selangor, Malaysia,

[6] Qaiwan International University, Faculty of Health Sciences, Sulaymaniyah, Iraq,

[7] Kurdistan Institution for Strategic Studies and Scientific Research (KISSR), Sulaymaniyah, Iraq,

[8] Department of Community Health, Universiti Putra Malaysia, 43400, Serdang, Selangor, Malaysia.

*Corresponding author

drrahmat@upm.edu.my

Abstract

Introduction

Wireless communication, connecting billions worldwide, relies on radiofrequency electromagnetic fields (RF-EMF). The advent of fifth-generation (5G) networks shifts RF carriers to higher frequencies. While radio, cell phones, and television have served human needs for decades, higher carrier frequencies pose potential health risks. Insects closely associated with humans, like mosquitoes, may experience increased RF absorption and dielectric heating. This could inadvertently impact their behavior, morphology, and physiology, possibly influencing their spread. This study investigates RF exposure effects on *Aedes aegypti* mosquitoes, prevalent in indoor environments with higher RF exposure risk. Our focus is on how RF exposure influences egg morphology and *Ae. aegypti* development.

Methods

In the experiment, triplicates of 30 eggs were exposed to RF radiation at baseline, 900 MHz, and 18 GHz. Daily observations in an insectarium tracked hatching responses, development times, larval numbers, and pupation periods until adult emergence.

Results

Results reveal RF radiation affecting the duration of mosquito growth from egg hatching to adult emergence and, although no significant differences in exposure levels were observed, some changes in egg morphology were noted.

Conclusion

This work highlights the sensitivity of *Ae. aegypti* egg morphology and development during the aquatic phase to RF radiation, potentially altering their life cycle.

Keywords: Aedes, larval development, radiation, radiofrequency, RF

Introduction

Mosquitoes hold significant public health importance as major vectors of diseases that affect human populations worldwide, with a particular focus on Aedes species. Among these, Aedes mosquitoes, responsible for transmitting dengue fever, are diurnal insects thriving in tropical and subtropical regions [1]. They prefer habitats in close proximity to humans and excel as vectors of arboviruses. A prominent dengue vector, *Aedes* (Stegomyia) *aegypti* (Linnaeus) (Diptera: Culicidae), is predominantly found in urban settings worldwide. The development rate of Aedes mosquitoes varies significantly due to diverse environmental factors, both biotic and abiotic [2]. The conjunction of climate, geography, environment, and social conditions creates an ideal environment for the survival of this vector species and the dengue virus. *Aedes aegypti*, commonly known as the 'yellow fever mosquito' [3], plays a pivotal role in transmitting dengue, chikungunya, and Zika viruses. Mosquitoes feature a complex life cycle closely intertwined with human environments. They are anthropophilic, exhibiting a strong association with human habitats and readily infiltrating buildings for feeding and resting. *Ae. aegypti* females lay their eggs in containers holding clear water, often with a minimal organic presence, positioned on moist surfaces just above the water level. Each female can lay approximately 100 eggs, repeating this process thrice in her lifetime [4]. These oval-shaped eggs, measuring around 1 mm in length, are slightly larger than those of *Ae. albopictus*. A comprehensive understanding of mosquito life cycles is crucial for unravelling insights into population biology, dynamics, vector capacity, and their impact on the risk of disease outbreaks.

Wireless communication, connecting billions of people globally, relies on radiofrequency electromagnetic fields (RF-EMF). The introduction of fifth-generation (5G) networks involves a partial shift of carrier frequencies to higher ranges. 5G networks can operate at frequencies reaching 300 GHz, nearing the millimeter wave frequency range, while

current telecommunications networks typically use frequencies between 0.1 and 6 GHz [5]. At these higher frequencies, the wavelength of the radiation becomes comparable to the size of an insect's body [6]. Ongoing research explores the potential effects of RF radiation on various living organisms, although its impact on wildlife remains largely unknown. Existing evidence suggests that environmental exposure to magnetic fields from RF, particularly in urban areas and near base stations, may affect the development and survival of certain species [7]. Experimentally, the effects of RF exposure on insects have yielded significant findings. Various species, including fruit flies exposed to 900 MHz RF radiation, have shown reduced reproductive capacity [8]. Likewise, [9] revealed colony weakening and disrupted returning ability in honeybees, and [10] reported disturbed cockroach navigation and migration. Furthermore, RF exposure has been linked to disruptions in the sense of smell in ants [11]. [10] also suggested that radiation from telecommunication antennas impairs honeybee navigation due to the effect on magnetite structures in the bee's body, which function as a natural compass. Additionally, other studies have highlighted the impact of RF exposure on microbial communities [12]. In the case of *Ae. aegypti*, research on collective positional behavior under relatively low-power RF exposure showed inconsistent effects on flight ability and direction [13]. A different study focusing on higher-frequency RF exposure found that it led to dielectric heating affecting the physiology, behavior, and morphology of adult *Ae. aegypti* [14].

While scientific evidence highlights the detrimental impact of RF exposure on various insect taxa, our understanding of the consequences of anthropogenic RF radiation on indoor-adapted mosquitoes, such as *Ae. aegypti*, remains limited. Particularly, the influence of RF exposure on the development of Aedes mosquito populations, especially *Ae. aegypti*, has not been explored. Such effects could potentially shape the proliferation of Aedes populations and impact the intensity of dengue transmission. In this study, our objective was to investigate the

impact of RF exposure on the life cycle of *Ae. aegypti*. Aedes species undergo holometabolous development, encompassing distinct stages from egg to larva, pupa, and adult. Therefore, we aimed to assess the influence of RF radiation on *Ae. aegypti's* developmental characteristics, from the early stages through to the emergence of adults.

Methods

Collection of mosquito strain

The laboratory strain of *Ae. aegypti* eggs was obtained from the Department of Medical Entomology, Infectious Diseases Research Centre, Institute of Medical Research in Kuala Lumpur, Malaysia. Field strain eggs were collected over a 16-week period, from March 2022 to June 2022, using ovitrap surveillance equipment placed in selected and strategically located sites with known established Aedes populations in Puncak Alam, Selangor [15-16]. The collection of *Ae. aegypti* followed the procedures described by [17]. Ovitraps were constructed using black plastic containers featuring a 4 cm-wide central opening, with dimensions of 8 cm in diameter and 11 cm in height. These ovitraps were reinforced with a 14 cm x 3 cm paddle and utilized as oviposition sites for Aedes females. Approximately two-thirds of the ovitraps were filled with water.

Colonization and mosquito cohort establishment

Field and laboratory strains of mosquitoes were reared and maintained in the vector insectary at the Faculty of Health Sciences, UiTM Puncak Alam, under controlled conditions of 29 ± 3°C, 75 ± 10% relative humidity (RH), and a 12:12-hour light-dark cycle. For field strains, eggs laid on the ovitrap paddle were submerged in containers filled with deionized water, allowing them to hatch. Approximately 4-7 days after hatching, 3-5 randomly selected

larvae were sampled for identification using a pipette. To ensure accurate taxonomic identification, larvae were briefly euthanized in hot water, placed on a glass slide, covered with a coverslip, and examined under a stereomicroscope. Aedes species were identified at the larval stage, with care taken to avoid disfigurement in handling deceased larvae. Identification relied on key taxonomic features, including a short, stout syphon, a row of thorn-like crest scales on abdominal segment VII, and the absence of hooks on the side of the thorax, as described by [18] and [19].

Aedes aegypti eggs were placed in deionized water enriched with nutrients, and larval growth was monitored daily. Fine fish food (TetraMin Plus Tropical Fish Flakes) was provided to support the growth of newly hatched larvae. To maintain optimal conditions and prevent foaming and volume loss in the tanks, daily water changes were conducted. To obtain virgin mosquitoes, sex separation was performed at the pupal stage, capitalizing on the size difference between female and male pupae. Batches of fifty pupae were segregated in plastic containers covered with fine mesh and containing 10 ml of dechlorinated water. For mating, 15 pairs of virgin adult mosquitoes were placed in standard rearing cages (30 x 30 x 30 cm). A 10% sucrose solution was provided as a nutrient source. After a 72-hour mating period, a blood meal was administered by introducing a confined white mouse into the mosquito cage for 12 hours to ensure that the female mosquitoes were sexually mature and had laid eggs. Following the blood meal, an egg-laying site was prepared within the cage using plastic containers (9 cm in height and 7.5 cm in diameter) filled with 100 ml of water and filter paper as the substrate. Strips with eggs were collected and replaced daily for one week. This cycle of larval development, adult mating, and oviposition was repeated until the F2 generation was obtained for both the laboratory and field strains. Only F2 generation mosquitoes were used for the study to determine if desired traits remained consistent in consecutive generations or exhibited unexpected changes [20]. After collection, eggs were allowed to embryonate for two days,

followed by drying on paper towels for 30 seconds and subsequent storage in a moist environment for an additional day. The eggs were then dried at rearing temperatures until they were slightly moist to the touch, not completely dry, and stored in airtight bags with moist absorbent cotton to maintain high humidity and prevent desiccation. A total of 1620 eggs from both the laboratory and field strains were required to conduct a single triplicate experiment on the effects of rapid, extreme RF exposure on egg hatching and larval development in *Ae. aegypti*.

Configuration of the exposure chamber and antenna

The experimental chamber comprises a large polystyrene foam box with dimensions of 49.5 cm x 62 cm x 37 cm and a thickness of approximately 3 cm (Fig 1). The entire chamber is uniformly painted white to enhance the contrast of the samples within. It consists of seven key compartments: (A) power supply, (B) analog signal generator, (C) spectrum analyzer, (D) RF antenna, (E) exposure chamber, (F) sample, and (G) RF cable. We utilized wideband antennas with frequency-dependent radiation characteristics, specifically the (A-Info, LB8180-NF model, J203020295; Irvine, CA, USA). This antenna boasts a frequency range of 0.8-18 GHz, an average gain of 12 dB, and linear polarization. It weighs approximately 1.5 kg and was employed to transmit the generated RF waves to the test subject. Due to its wide bandwidth, a single antenna suffices to radiate power across various frequencies.

As depicted in Fig 1, the antenna was positioned within the designated compartment and oriented towards the intervention site. The source of the RF wave for this study is a wideband function generator (Keysight, model N5173B EXG X-Series; Colorado Springs, CO, USA) capable of producing high-frequency signals ranging from 9 kHz to 40 GHz. The signal generator is linked to a super ultra-wideband amplifier through a coaxial cable with SMA

connectors to enhance the signal. The amplifier is then connected to the antenna via a coaxial cable, with both cables terminated with SMA-to-N connectors.

Fig 1. **Designated chamber and antenna of RF exposure on *Ae. aegypti* eggs.**

RF Exposure Protocols

The eggs of the F2 generation *Ae. aegypti* strain were meticulously inspected for defects under a stereomicroscope (Olympus, model Courtesy; UK Ltd) before being placed on filter paper in plastic containers (16.5 cm x 7.5 cm x 10 cm) with lids and kept at room temperature. Ninety mosquito eggs were subjected to separate exposures of two different RF frequencies, namely, (a) 900 MHz and (b) 18 GHz, each lasting for 24 hours in dedicated chambers. Each RF exposure procedure was repeated three times (in triplicate) to ensure the validity and reliability of results for both the laboratory and field strains of *Ae. aegypti*. As for control, the procedures were duplicated without any RF exposure. Maintaining a single RF exposure for *Ae. aegypti* eggs is essential to assess mosquito development.

Experimental design

In this experimental design, a completely randomized factorial 2 x 2 x 3 design was employed, resulting in a total of 12 treatments. The independent variables consisted of two levels of exposure duration: a 24-hour exposure and a control group with no exposure (Table 1). Additionally, the design incorporated variations in the *Ae. aegypti* population as a separate factor, which could potentially be influenced by the exposure duration. To ensure the robustness of the study's findings, three replications were included, involving the repetition of the entire experiment three times. Each combination of these independent variables represented a unique treatment condition, resulting in a diverse set of 12 treatments. The use of a completely

randomized design ensured that treatments were assigned to experimental units in a random and unbiased manner, enhancing the reliability and validity of the results. This design facilitated a systematic and controlled examination of how exposure duration and population variations impact the *Ae. aegypti* population, ultimately enhancing the study's comprehensiveness and reliability.

For the egg weighing analysis, a total of 30 eggs from both the field and laboratory strains of *Ae. aegypti* were used for each replicate, including unexposed (control), 900 MHz, and 18 GHz groups. In each session, all the eggs were placed in 1.5 ml Eppendorf tubes and weighed using a high-precision electronic microbalance scale (Sartorius, model MPR 5; Goettingen, Germany) with an accuracy of 0.0001 g. To assess the sample's variation after RF exposure, average weights were computed by weighing the entire sample of eggs. The egg morphology of *Ae. aegypti* was assessed by examining the eggs under an inverted microscope (Olympus, model IX73; UK Ltd). Images of the eggs were captured, transferred to a computer, and photographed using an image documentation system, DinoCapture 2.0 [21]. The morphometric measurements were analyzed with Image J software [22-23]. Morphological parameters, including length, width, egg index, and surface area, were evaluated. At least nine eggs from each RF exposure level and control were measured using Image J. Egg length was determined as the distance between the anterior and posterior poles. Egg width was measured at the widest point [24]. The egg index was calculated based on the length-to-width ratio [25], and surface area was automatically estimated using Image J.

The aquatic phase of larval development was observed daily under controlled conditions at 29 ± 3°C, 75 ± 10% relative humidity, and a 12:12 hours light-dark cycle. Within the scope of this study, observations included assessing the egg hatching rate, larval mortality, pupation rate, pupation duration, pupal onset (CP1) and termination (CP2), and adult emergence rate. The rearing temperature and conditions were standardized at room

temperature. When the eggs began to hatch, a small amount of TetraMin Plus Tropical Fish Flakes was introduced into the tank. Larvae were adequately fed, and water clarity was maintained through regular water changes to provide optimal conditions for larval growth. Typically, the first pupae emerged around day 6-7, but they were left undisturbed in the tank for over 24 hours to ease the separation process from the larvae. During the pupation process, the pupae should be transferred to a labelled plastic cup (name, pupation date) with clean water and mesh cover using plastic pipettes. Feeding of larvae must continue, but the amount of food should be reduced once pupation is observed to avoid excessive contamination and foaming. The adults were fed 10% sucrose, and on the second day after adult hatching, the adult *Ae. aegypti* were aspirated with a suction cup and placed in a labelled universal bottle. This bottle was placed in the refrigerator at 4°C for two hours after removal of the adults to kill the adults.

Table 1. Experimental design table.

**Factors**		**Replicate of experiment, no of eggs**			**Total no of eggs**
**Field strain**	**Lab strain**	**I**	**II**	**III**	
(-) [a]	(-) [a]	30	30	30	90
(+) [b]	(-) [a]	30	30	30	90
(-) [a]	(+) [b]	30	30	30	90
(+) [b]	(+) [b]	30	30	30	90
**Total eggs required / strain**					**360**

The experiment was completely randomized in a factorial 2 x 2 x 3 design (12 treatments). Independent variables consisted of two exposures duration (24 hours and control) on *Ae. aegypti* population and three replications

[a] Symbol (-) refer to the eggs that were unexposed (control) with RF radiation

[b] Symbol (+) refer to the eggs that were exposed with RF radiation (24 hours)

Data analysis

The database of variables was analyzed using IBM SPSS Statistics (version 25, copyright IBM Corporation). Parametric analysis with paired t-tests was employed to determine whether statistically significant differences existed between the mosquitoes irradiated with RF and the control mosquitoes for both the laboratory and field strains. This test aimed to establish whether there were any changes in *Ae. aegypti* following exposure to radiation during the immature phase (eggs). In cases where the paired *t-test* revealed a significant difference between mosquito samples (control, laboratory strain, field strain), a Tukey or Dunnett's T3 post-hoc test was conducted. The results of the paired t-test analysis, with a significance level set at 0.05, were evaluated and discussed.

Ethics statement

The study was approved by the Committee on Animal Research and Ethics (UiTM CARE) under the reference number UiTM CARE: 377/2022(13th May 2022). The approval duration was July 2022 until June 2023.

Results

Effect of RF exposure on egg weight of *Ae. aegypti*

The tabulated results offer valuable insights into the influence of RF radiation exposure on the weight of *Ae. aegypti* mosquito eggs. Within Table 2, the mean egg weight is categorized based on different strains of *Ae. aegypti* subjected to varying RF conditions, including control, 900 MHz, and 18 GHz exposures. Consistently, the results indicate that the mean egg weight of the laboratory strain surpasses that of the field strain across all conditions. For instance, in the control group, the egg weight of the laboratory strain measured 12.40 µg ± 0.61, whereas

the field strain recorded 11.97 ± 1.43 µg. Likewise, in groups exposed to 900 MHz and 18 GHz radiation, the eggs of the laboratory strain (12.60 ± 0.40 µg and 12.23 ± 1.90 µg) exhibited greater weight compared to those of the field strain (11.37 ± 0.56 µg and 11.33 ± 0.65 µg). These findings strongly suggest that the laboratory strain may possess a higher level of resilience to the effects of RF exposure.

Table 2. Egg weight based on different strains of *Ae. aegypti*.

Exposure	Strain	I	II	III	Mean	±SD
**Control**	Laboratory	13.10	12.11	11.98	12.40	±0.61
	Field	10.33	12.66	12.93	11.97	±1.43
**900 MHz**	Laboratory	12.56	13.01	12.22	12.60	±0.40
	Field	11.25	11.98	10.89	11.37	±0.56
**18 GHz**	Laboratory	14.01	10.22	12.45	12.23	±1.90
	Field	10.67	11.97	11.34	11.33	±0.65

Mean weight (mg) and standard deviation (SD) for laboratory strain and field strain of *Ae. aegypti* for different doses exposure of RF radiation.

Effect of RF radiation exposure on egg morphology of *Ae. aegypti*

Table 3 provides a comprehensive overview of *Ae. aegypti* egg morphology, including length (µm), width (µm), area (µm²), and egg index (µm), following exposure to RF radiation at both 900 MHz and 18 GHz. The findings reveal noteworthy distinctions between the control and RF-exposed eggs from laboratory and field strains. In general, the study emphasizes that eggs from the control group of the laboratory strain consistently displayed larger dimensions in terms of length, width, area, and egg index compared to the eggs from the field strain. Furthermore, laboratory eggs exposed to RF radiation at both 900 MHz and 18 GHz exhibited elongated lengths, larger areas, and higher egg indices when compared to field eggs exposed

to the same RF conditions. Interestingly, field strain eggs subjected to RF radiation at 900 MHz and 18 GHz displayed greater width dimensions than their laboratory counterparts. For a more detailed examination of the measurements, the study revealed the following results: Control group eggs from laboratory strains had an average length of 745.63 ± 19.82 µm, a width of 202.42 ± 16.65 µm, an area of 751.80 ± 23.75 µm², and an egg index of 3.70 ± 0.24 µm. In contrast, eggs from field strains in the control group exhibited an average length of 672.71 ± 36.13 µm, a width of 200.14 ± 35.26 µm, an area of 703.20 ± 62.13 µm², and an egg index of 3.47 ± 0.70 µm. Furthermore, laboratory strain eggs exposed to 900 MHz radiation had an average length of 726.87 ± 23.34 µm, a width of 194.32 ± 19.48 µm, an area of 726.87 ± 23.34 µm², and an egg index of 3.76 ± 0.40 µm. When exposed to 18 GHz radiation, laboratory strain eggs displayed an average length of 726.89 ± 34.52 µm, a width of 198.88 ± 27.49 µm, an area of 727.92 ± 36.17 µm², and an egg index of 3.71 ± 0.50 µm.

On the other hand, field strain eggs exposed to 900 MHz radiation exhibited an average length of 702.21 ± 61.59 µm, a width of 211.02 ± 52.44 µm, an area of 678.35 ± 38.58 µm², and an egg index of 3.56 ± 1.09 µm. When exposed to 18 GHz radiation, field strain eggs displayed an average length of 689.49 ± 18.98 µm, a width of 209.29 ± 26.21 µm, an area of 685.14 ± 20.55 µm², and an egg index of 3.34 ± 0.40 µm. These detailed measurements provide insights into the intricate variations in egg morphology resulting from RF radiation exposure in laboratory and field strains of *Ae. aegypti*. Table 4 presents the results of a morphometric analysis comparing two distinct groups of RF radiation. In both laboratory and field strains, it can be concluded that there were no significant differences observed in the measurements of egg length, width, area, and index among all the results ($p>0.05$). These findings suggest an absence of a correlation between unexposed and RF-exposed eggs, with no discernible correlation between low-level RF exposure (900 MHz) and high-level RF exposure (18 GHz).

Table 3. Egg morphology measurements based on different strains of *Ae. aegypti*.

Exposure	Strain	Length (µm)	Width (µm)	Area (µm²)	Egg Index (µm)
**Control**	Laboratory	745.63 ±19.82	202.42 ±16.65	751.80 ±23.75	3.70 ±0.24
	Field	672.71 ±36.13	200.14 ±35.26	703.20 ±62.13	3.47 ±0.70
**900 MHz**	Laboratory	726.87 ±23.34	194.32 ±19.48	726.87 ±23.34	3.76 ±0.40
	Field	702.21 ±61.59	211.02 ±52.44	678.35 ±38.58	3.56 ±1.09
**18 GHz**	Laboratory	726.89 ±34.52	198.88 ±27.49	727.92 ±36.17	3.71 ±0.50
	Field	689.49 ±18.98	209.29 ±26.21	685.14 ±20.55	3.34 ±0.40

Note: Mean egg length (µm) , width (µm), area (µm²) and egg index (µm) with standard deviation (SD) for laboratory strain and field strain of *Ae. aegypti* for different doses exposure of RF radiation

Table 4. Comparison of egg morphometric analysis based on radiofrequency for both laboratory and field strain of *Ae. aegypti.*

Strain	Parameter	Exposure	*p*-value
**Laboratory strain**	Length (µm)	Control and 900 MHz	0.12
		Control and 18 GHz	0.28
		900 MHz and 18 GHz	0.83
	Width (µm)	Control and 900 MHz	0.31
		Control and 18 GHz	0.73
		900 MHz and 18 GHz	0.68
	Area (µm²)	Control and 900 MHz	0.10
		Control and 18 GHz	0.22
		900 MHz and 18 GHz	0.93
	Egg index	Control and 900 MHz	0.61
		Control and 18 GHz	0.94
		900 MHz and 18 GHz	0.80
**Field strain**	Length (µm)	Control and 900 MHz	0.21
		Control and 18 GHz	0.31
		900 MHz and 18 GHz	0.55
	Width (µm)	Control and 900 MHz	0.45
		Control and 18 GHz	0.38
		900 MHz and 18 GHz	0.88
	Area (µm²)	Control and 900 MHz	0.29
		Control and 18 GHz	0.70
		900 MHz and 18 GHz	0.43
	Egg index	Control and 900 MHz	0.75
		Control and 18 GHz	0.58
		900 MHz and 18 GHz	0.44

Effect of RF radiation exposure on egg hatching of *Ae. aegypti*

This section provides insights into the results of an experiment that assessed the hatching response of *Ae. aegypti* eggs from laboratory strains following exposure to various types of RF radiation. As depicted in Fig. 2, the hatching response of *Ae. aegypti* post-RF irradiation is illustrated. In Fig. 2A, the graph demonstrates that all three radiation exposures resulted in hatching rates exceeding 50%. The control group displayed the lowest hatching rate at 61.67 ± 29.87%, followed by the 18 GHz group with a rate of 68.67 ± 14.74%. The highest hatching rate was observed in the 900 MHz exposure group, registering at 79 ± 10.54%. However, the data indicate that there were no significant differences in hatching rates between the control group, the 900 MHz exposure group, and the 18 GHz exposure group (all $p > 0.05$).

Fig. 2B presents the results of an experiment that assessed the hatching duration of *Ae. aegypti* eggs from a laboratory strain after exposure to different levels of RF radiation. The graph illustrates that the mean hatching time for the group exposed to 18 GHz radiation was the shortest, averaging 5.5 ± 3.61 days, followed by the group exposed to 900 MHz, with a mean hatching time of 6 ± 3.89 days. In contrast, the control group, which was not exposed to RF radiation, had the longest mean hatching time, standing at 6.5 ± 4.18 days. Furthermore, the results provide insights into the range of hatching times for each RF radiation exposure. The group exposed to 18 GHz radiation demonstrated the fastest hatching times, with a range from a minimum of less than 24 hours to a maximum of 11 days. Similarly, the 900 MHz exposure group displayed hatching times ranging from a minimum of less than 24 hours to a maximum of 12 days. The control group exhibited hatching times that ranged from a minimum of less than 24 hours to a maximum of 11 days. The boxplot featured in the figure emphasizes that exposure to RF-EMF leads to shorter hatching durations compared to unexposed eggs (control).

Fig 2. **Egg hatching of *Ae. aegypti*.** A. Hatching rate (%) and B. Hatching period (day) of *Ae. aegypti* eggs after being exposed with RF radiation.

Effect of RF radiation exposure on the development of *Ae. aegypti*

Fig. 3 presents the results of an experiment that examined the developmental stages of *Ae. aegypti*, ranging from the larval phase to adulthood, under varying RF radiation exposures. In Fig. 3A, a comparison is made regarding the onset of the larval phase among the control group, the 900 MHz exposure group, and the 18 GHz exposure group. Both the control and 900 MHz groups exhibited a similar proportion of eggs hatching into the first larval stage in under 24 hours (8% and 44%, respectively). Conversely, the 18 GHz group had an onset on the first day, with 6% of eggs hatching into the first larval stage. The control group displayed the swiftest larval development, taking 9 ± 1.73 days to progress to the pupal stage, compared to 9.33 ± 0.76 days for the 900 MHz group and 9.83 ± 0.58 days for the 18 GHz group. Notably, the group exposed to 18 GHz completed the emergence of adult *Ae. aegypti* at 14.23 ± 1.04 days, whereas the 900 MHz group and the control group reached this stage at 14.56 ± 0.97 days and 14.78 ± 2.66 days, respectively. It's essential to mention that the 900 MHz group did not achieve a 50% adultization rate.

Fig. 3B reveals the adultization rate, with the control group registering the highest rate, followed by the 18 GHz group and the 900 MHz group at 68%, 50%, and 33%, respectively. In terms of pupation time, as shown in Fig. 3C, the onset of pupation (CP1) remained consistent across all groups, taking 7 ± 1 days. Concerning the completion of pupation (CP2), the control group exhibited the shortest duration at 18 ± 1.58 days, followed by the 900 MHz group at 21.33 ± 1.53 days and the 18 GHz group at 22.5 ± 1.29 days. The boxplot underscores that lower RF radiation exposure correlates with shorter pupation times for immature *Ae. aegypti* in their transformation into adult mosquitoes.

Fig 3. **Development stage of *Ae. aegypti* from larva stage to adulthood.** A. The development trend of immature exposed to different RF radiation (control, 900 MHz, 18 GHz) (%); B. adult emergence (%); C. commencement and cessation period of pupation (days).

Discussion

The ongoing global resurgence of vector-borne diseases, exacerbated by rapid urbanization and high population density, has resulted in the widespread occurrence of conditions such as dengue fever, Zika, yellow fever, and chikungunya. One of the primary vectors responsible for the transmission of these diseases, particularly well-adapted to urban environments, is the *Ae. aegypti* mosquito. This study was conducted to explore the effects of RF radiation exposure on the life cycle and characteristics of *Ae. aegypti*, with a particular focus on egg morphology and development.

Our findings consistently revealed that the laboratory strain of *Ae. aegypti* consistently exhibited higher average egg weights than the field strain across all experimental conditions. The variation in average egg weight may be attributed to differing levels of radiation exposure, with the field strain eggs experiencing a slight decrease in weight between the control, 900 MHz, and 18 GHz exposure groups. This trend suggests that the laboratory strain may possess greater resilience to the effects of RF radiation. Furthermore, our study suggests a potential relationship between RF radiation exposure and the average weight of mosquito eggs. It is important to note that the differences in egg weight measurements could be associated with the natural process in which freshly laid *Ae. aegypti* eggs gradually absorb water over the initial 16 hours, maintaining a stable weight until the completion of embryogenesis, as previously reported [26]. Additionally, *Ae. aegypti* eggs are known to be susceptible to water loss, which can introduce fluctuations in their weight and potentially impact egg viability, as has been

discussed in prior studies [27]. It is crucial to acknowledge that there is a lack of information regarding the typical egg weight of mosquitoes, and previous research predominantly focused on different species, such as *Ae. albopictus*, with an emphasis on resistance to desiccation. This context makes direct comparisons between our study's outcomes and those of previous research challenging. Nevertheless, this study represents an important initial step that may serve as a foundation for future investigations into the typical egg weight of mosquitoes and the potential implications of RF radiation exposure on this fundamental aspect of their biology.

This study has brought to light significant distinctions in egg length between laboratory and field strains of *Ae. aegypti*, both in the absence of RF radiation exposure and following exposure to 900 MHz and 18 GHz radiation. Notably, eggs from the laboratory strains consistently exhibited greater length measurements compared to those of field strains, regardless of RF exposure. However, unexposed field strains displayed longer eggs than their laboratory counterparts subjected to radiation. Intriguingly, egg length exhibited relative consistency between the laboratory strain exposed to 18 GHz radiation and the non-irradiated eggs. These measurements closely align with the findings of a study by 24 while deviating from results reported by [25] and [28]. These disparities may be attributed to variations in geographical origin, topographical features of the study sites, diverse climatic conditions, and other contributing factors. Of particular interest, this investigation unveiled that eggs from both exposed and unexposed laboratory and field strains exhibited narrower dimensions in contrast to previous studies conducted by [25], [29], and [24]. Furthermore, the study yielded egg index values that closely resembled the findings of [25]. Notably, these studies encompassed diverse geographic locations, including Australia and Brazil, highlighting the potential influence of geographical origin on the morphological characteristics of mosquito eggs.

The study unveiled a significant impact of RF radiation exposure on the hatching process of mosquito eggs. Notably, eggs exposed to high-intensity RF at 900 MHz and 18 GHz

exhibited the highest hatching rates when compared to the control group. Intriguingly, these eggs also hatched relatively quickly. Conversely, eggs exposed to the lower dose of 900 MHz radiation demonstrated the highest average hatching rate but required a more moderate amount of time to hatch. In contrast, the control group, which remained unexposed to RF radiation, displayed a moderate hatching rate but took the longest time to complete the hatching phase. It's noteworthy that RF exposure directly influenced both the average hatching rate and the duration of hatching. Specifically, the 900 MHz radiation and non-exposure conditions resulted in a slower hatching process compared to the 18 GHz radiation exposure, which led to shorter hatching times.

These findings align with previous research conducted by [30] and [31], both of whom observed a significant decrease in hatching rates with increasing X-ray exposure. Furthermore, the study's researchers delved into the impact of pupal irradiation using 40 Gy and 60 Gy of RF from cell phone radiation on the sterility and pupal development of honey bees. When normal males were paired with adult females derived from irradiated pupae, mating outcomes, including egg production and hatching rates, were notably inferior compared to control groups. Moreover, they uncovered a positive correlation between the radiation dose and the quantity of eggs and hatching rates, as demonstrated by [32]. Additionally, other studies, such as the one conducted by [10], demonstrated that the use of GSM with 900 MHz radiation from cell phones led to reduced bee strength and a lower queen egg laying rate. Exposed queens in test colonies produced fewer eggs per day compared to control queens. These outcomes could be attributed to the damaging effects of RF radiation on cells and DNA, potentially resulting in aberrant development or even embryo mortality. It's crucial to note that these findings provide a general understanding, and the hatching rates of Aedes eggs may vary based on the specifics of the studies and experiments conducted.

This study also unveiled that varying RF radiation exposure had a noticeable impact on the developmental abilities of this species. The unexposed group exhibited the lengthiest development time from egg hatching to adult emergence, in contrast to the groups exposed to 900 MHz and 18 GHz radiation. Interestingly, while the 900 MHz radiation-exposed group demonstrated the fastest development from the egg to the larval stage during the aquatic phase, it also had the lowest rate of adult emergence compared to both the control group and the 18 GHz group. These findings align with previous research on Ae. albopictus development and the effects of electromagnetic field (EMF) exposure. [33] observed that exposure to microwave EMF led to a delay in pupation, while having no impact on the pupation rate. In contrast, [34] noted that, despite a decrease in the pupation rate with increasing gamma irradiation dose in *Ae. aegypti* mosquitoes, there was no statistically significant difference between the control group and the 70 Gy dose. A similar conclusion was reached by [22], who found that the dose of gamma irradiation administered to *Ae. aegypti* pupae had no statistically significant effect on the percentage of successfully hatched adults. Furthermore, [31] reported that, across all age groups and X-ray irradiation doses, pupal hatching rates of *Ae. albopictus* were not significantly different from the control group.

The intriguing trend observed in this study is that eggs of *Ae. aegypti* exposed to RF irradiation exhibited a different developmental pattern compared to non-irradiated eggs. Notably, adult development was faster in eggs exposed to 900 MHz radiation compared to those unexposed to RF radiation or exposed to 18 GHz radiation. It's plausible that the effects of RF radiation on egg physiology and biochemistry may influence egg growth rates, contributing to this pattern, as suggested by [34]. Furthermore, it's conceivable that radiation from RF has an impact on the physiology and biochemistry of both adult mosquitoes and larvae, which could also affect developmental rates. However, it's imperative to emphasize that further

research is essential to gain a comprehensive understanding of the complete effects of RF radiation on insect development and the underlying mechanisms at play.

Conclusion

Understanding the impact of radiofrequency (RF) exposure on Aedes mosquitoes, with a particular focus on *Ae. aegypti*, holds immense significance in the realm of dengue fever control due to the pivotal role this species plays as a primary disease vector. Our research has revealed that higher doses of RF radiation can expedite the hatching process of Aedes mosquitoes, while concurrently slowing down their overall developmental rate. These findings constitute a foundational step towards a more profound comprehension of how RF radiation influences Aedes mosquito populations, furnishing invaluable insights into the dynamics of these populations. However, it is imperative to acknowledge the potential variability in results under distinct study conditions, encompassing variations in the type of RF exposure and the mosquito species studied. Consequently, further research is not just desirable but essential to holistically unravel the ramifications of RF exposure on *Aedes albopictus* and to identify the most effective strategies for applying this knowledge in dengue vector control. The scientific community continues to explore the effects of RF exposure on insects, notably mosquitoes, as multiple studies have indicated that such exposure can impact hatching and developmental processes. It is abundantly clear that more comprehensive research is warranted to fully comprehend the scope of these effects and to elucidate their practical applications in efforts aimed at controlling dengue transmission. These ongoing studies are pivotal in comprehending the potential utility of RF exposure as a tool to mitigate the spread of dengue disease and in establishing the most efficacious approaches for translating this knowledge into practical control measures.

Availability of data and material

All data underlying the findings described are fully available without restriction.

Competing interests

The authors affirm that they have no known financial or interpersonal conflicts that would have appeared to have an impact on the research presented in this study.

Author Contributions

Conceptualisation: Nik Muhammad Hanif Nik Abdull Halim, Nazri Che Dom, Rahmat Dapari

Methodology: Nik Muhammad Hanif Nik Abdull Halim, Alya Farzana Mohd Jamili, Nazri Che Dom, Nurul Huda Abd Rahman, Rahmat Dapari.

Writing – original draft: Nik Muhammad Hanif Nik Abdull Halim, Alya Farzana Mohd Jamili, Nazri Che Dom, Nurul Huda Abd Rahman

Writing – review & editing: Nik Muhammad Hanif Nik Abdull Halim, Alya Farzana Mohd Jamili, Nazri Che Dom, Nurul Huda Abd Rahman, Zana Jamal Kareem, Rahmat Dapari

Acknowledgement

The authors wish to express their deep gratitude to the organizations that were instrumental in the completion of this project. A special note of appreciation goes to the Faculty of Health Sciences at Universiti Teknologi MARA (UiTM) for their invaluable technical support and guidance during this research project.

References

1. Nasir S, Asrar M, Rasul A, Hussain SM, Aslam N, Ahmed F, et al. Potential impact of global warming on population dynamics of dengue mosquito, *Aedes albopictus* Skuse (Diptera; Culicidae). Pakistan Journal of Agricultural Sciences. 2018;55(4).
2. Tomé HV, Pascini TV, Dângelo RA, Guedes RN, Martins GF. Survival and swimming behavior of insecticide-exposed larvae and pupae of the yellow fever mosquito *Aedes aegypti*. Parasites & Vectors. 2014; 7:1-9.
3. Sukiato F, Wasserman RJ, Foo SC, Wilson RF, Cuthbert RN. The effects of temperature and shading on mortality and development rates of *Aedes aegypti* (Diptera: Culicidae). Journal of Vector Ecology. 2019;44(2):264-70.
4. Kettle DS. Medical and veterinary entomology. Croom Helm Ltd; 1984.
5. Bhatt CR, Thielens A, Billah B, Redmayne M, Abramson MJ, Sim MR, et al. Assessment of personal exposure from radiofrequency-electromagnetic fields in Australia and Belgium using on-body calibrated exposimeters. Environmental Research. 2016; 151:547-63.
6. Pi Z, Khan F. An introduction to millimeter-wave mobile broadband systems. IEEE communications magazine. 2011;49(6):101-7.
7. Cucurachi S, Tamis WL, Vijver MG, Peijnenburg WJ, Bolte JF, de Snoo GR. A review of the ecological effects of radiofrequency electromagnetic fields (RF-EMF). Environment International. 2013; 51:116-40.
8. Panagopoulos DJ, Karabarbounis A, Margaritis LH. Effect of GSM 900-MHz mobile phone radiation on the reproductive capacity of *Drosophila melanogaster*. Electromagnetic Biology and Medicine. 2004 Jan 1;23(1):29-43.

9. Vácha M, Puzová T, Kvícalová M. Radio frequency magnetic fields disrupt magnetoreception in American cockroach. Journal of Experimental Biology. 2009;212(21):3473-7.
10. Sahib SS. Electromagnetic radiation (EMR) clashes with honey bees. International Journal of Environmental Sciences. 2011;1(5):897-900.
11. Cammaerts MC, De Doncker P, Patris X, Bellens F, Rachidi Z, Cammaerts D. GSM 900 MHz radiation inhibits ants' association between food sites and encountered cues. Electromagnetic Biology and Medicine. 2012;31(2):151-65.
12. Dev SR, Birla SL, Raghavan GS, Subbiah J. Microbial decontamination of food by microwave (MW) and radio frequency (RF). Microbial Decontamination in The Food Industry. 2012; 274-299. Woodhead Publishing.
13. Poh AH, Moghavvemi M, Shafiei MM, Leong CS, Lau YL, Mahamd Adikan FR, et al. Effects of low-powered RF sweep between 0.01-20 GHz on female *Aedes aegypti* mosquitoes: A collective behaviour analysis. PloS ONE. 2017;12(6): e0178766.
14. De Borre E, Joseph W, Aminzadeh R, Müller P, Boone MN, Josipovic I, et al. Radio-frequency exposure of the yellow fever mosquito (*Ae. aegypti*) from 2 to 240 GHz. PLOS Computational Biology. 2021 Oct 28;17(10): e1009460.
15. Awang MF, Dom NC. The effect of temperature on the development of immature stages of Aedes spp. against breeding containers. International Journal of Global Warming. 2020;21(3):215-33.
16. Shahrudin NA, Dom NC, Ishak AR. Temperature stress effect on the survival of *Aedes albopictus* (Skuse) (Diptera: Culicidae) adult mosquito: an experimental study. Malaysian Journal of Medicine & Health Sciences. 2019;15.

17. Faull KJ, Williams CR. Differentiation of *Aedes aegypti* and *Aedes notoscriptus* (Diptera: Culicidae) eggs using scanning electron microscopy. Arthropod Structure & Development. 2016;45(3):273-80.

18. Rueda LM. Pictorial keys for the identification of mosquitoes (Diptera: Culicidae) associated with dengue virus transmission. Zootaxa. 2004;589(1):1-60.

19. Christopher S. *Aedes Aegypti* (L.), the Yellow Fever Mosquito. Its life history, bionomics, and structure. Cambridge University Press, New York, 1960;133(3463):1473-4.

20. Alphey L. Genetic control of mosquitoes. Annual review of entomology. 2014; 59:205-24.

21. Othman AN, Suryani Zainudin NF, Zaidan UH, Shamsi S. A Comparative Study on the Larvicidal Effects of Piper sarmentosum (Kaduk) Leaves Extracts against *Aedes aegypti*. Pertanika Journal of Science & Technology. 2021;29(4).

22. Aldridge RL, Kline J, Coburn JM, Britch SC, Boardman L, Hahn DA, et al. Gamma-irradiation reduces survivorship, feeding behavior, and oviposition of female *Aedes aegypti*. Journal of the American Mosquito Control Association. 2020;36(3):152-60.

23. Mains JW, Mercer DR, Dobson SL. Digital image analysis to estimate numbers of Aedes eggs oviposited in containers. Journal of the American Mosquito Control Association. 2008;24(4):496.

24. Suman DS, Shrivastava AR, Pant SC, Parashar BD. Differentiation of *Aedes aegypti* and *Aedes albopictus* (Diptera: Culicidae) with egg surface morphology and morphometrics using scanning electron microscopy. Arthropod Structure & Development. 20111;40(5):479-83.

25. Mundim-Pombo AP, Carvalho HJ, Rodrigues Ribeiro R, León M, Maria DA, Miglino MA. *Aedes aegypti*: egg morphology and embryonic development. Parasites & Vectors. 2021;14(1):1-2.

26. Urbanski JM, Benoit JB, Michaud MR, Denlinger DL, Armbruster P. The molecular physiology of increased egg desiccation resistance during diapause in the invasive mosquito, *Aedes albopictus*. Proceedings of the Royal Society B: Biological Sciences. 2010;277(1694):2683-92.

27. Vargas HC, Farnesi LC, Martins AJ, Valle D, Rezende GL. Serosal cuticle formation and distinct degrees of desiccation resistance in embryos of the mosquito vectors *Aedes aegypti*, *Anopheles aquasalis* and *Culex quinquefasciatus*. Journal of Insect Physiology. 2014; 62:54-60..

28. Bova J, Paulson S, Paulson G. Morphological differentiation of the eggs of North American container-inhabiting Aedes mosquitoes. Journal of the American Mosquito Control Association. 2016;32(3):244-6.

29. Win MM, Swe TT, Sett KM, Mya MM, Latt AZ, Soe K, et al. Structural differentiation of *Aedes aegypti* and *Aedes albopictus* eggs using Scanning Electron Microscope. Journal of Biological Engineering Research and Review. 2018;5(1):09-12.

30. Ranathunge T, Harishchandra J, Maiga H, Bouyer J, Gunawardena YN, Hapugoda M. Development of the Sterile Insect Technique to control the dengue vector *Aedes aegypti* (Linnaeus) in Sri Lanka. PLoS ONE. 2022;17(4): e0265244.

31. Du W, Hu C, Yu C, Tong J, Qiu J, Zhang S, Liu Y. Comparison between pupal and adult X-ray radiation, designed for the sterile insect technique for *Aedes albopictus* control. Acta Tropica. 2019; 199:105110.

32. Odemer R, Odemer F. Effects of radiofrequency electromagnetic radiation (RF-EMF) on honey bee queen development and mating success. Science of the Total Environment. 2019; 661:553-62.

33. Atli E, Ünlü H. The effects of microwave frequency electromagnetic fields on the development of *Drosophila melanogaster*. International Journal of Radiation Biology. 2006;82(6):435-41.

34. Ernawan B, Anggraeni T, Yusmalinar S, Ahmad I. Investigation of developmental stage/age, gamma irradiation dose, and temperature in sterilization of male *Aedes aegypti* (Diptera: Culicidae) in a sterile insect technique program. Journal of Medical Entomology. 2022;59(1):320-7.

Figure 1

Click here to access/download;Figure;Fig 1.pdf

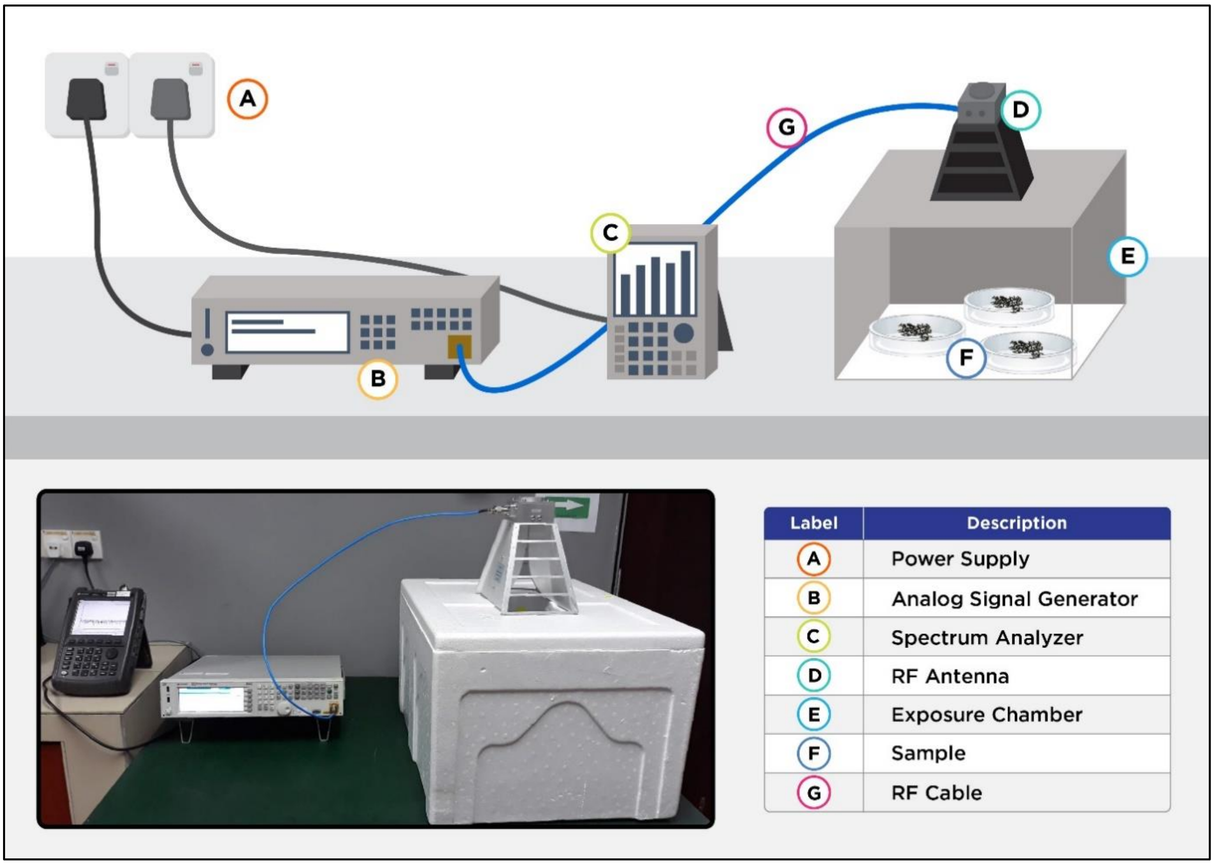

Click here to access/download;Figure;Fig 2.pdf

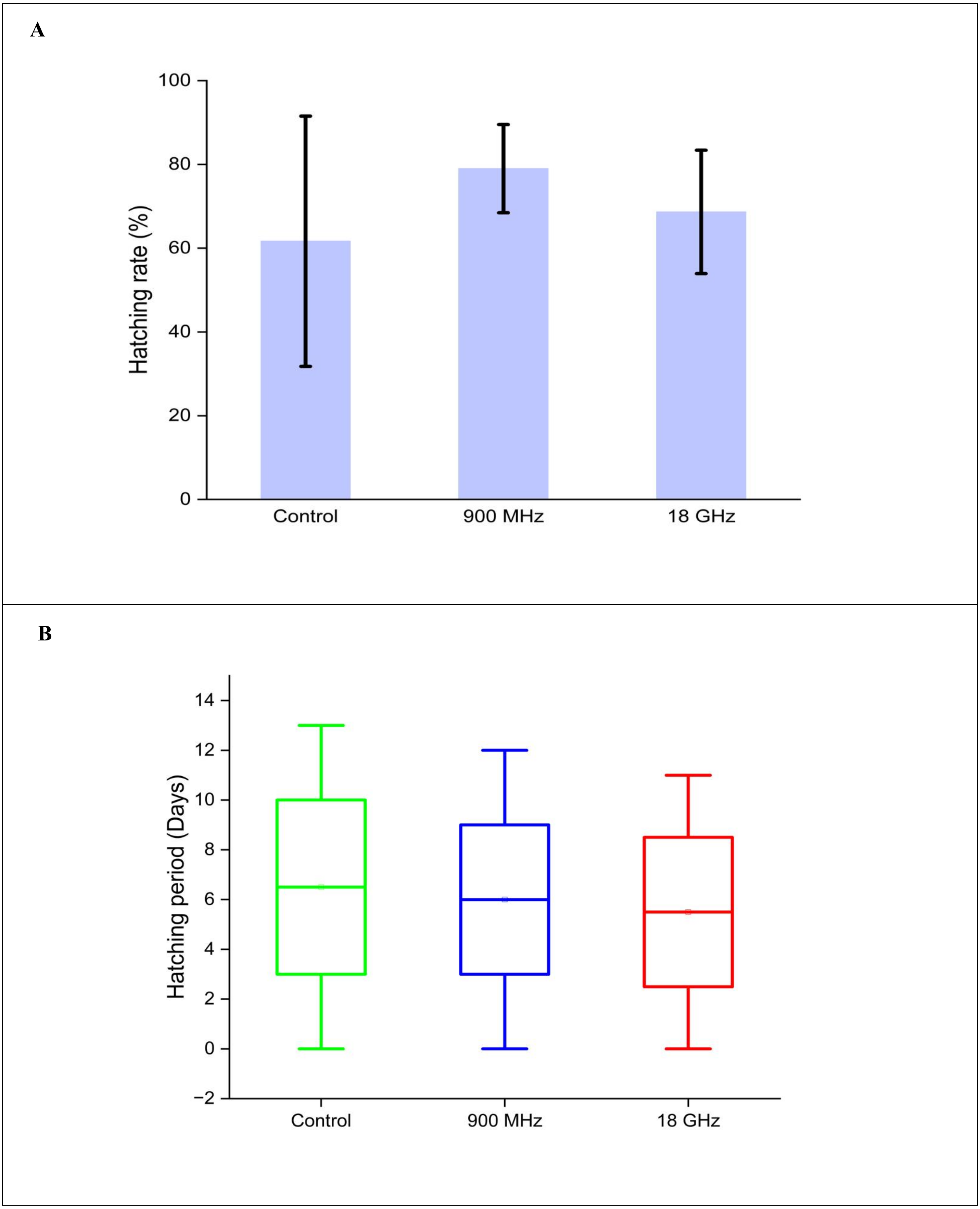

Click here to access/download;Figure;Fig 3.pdf

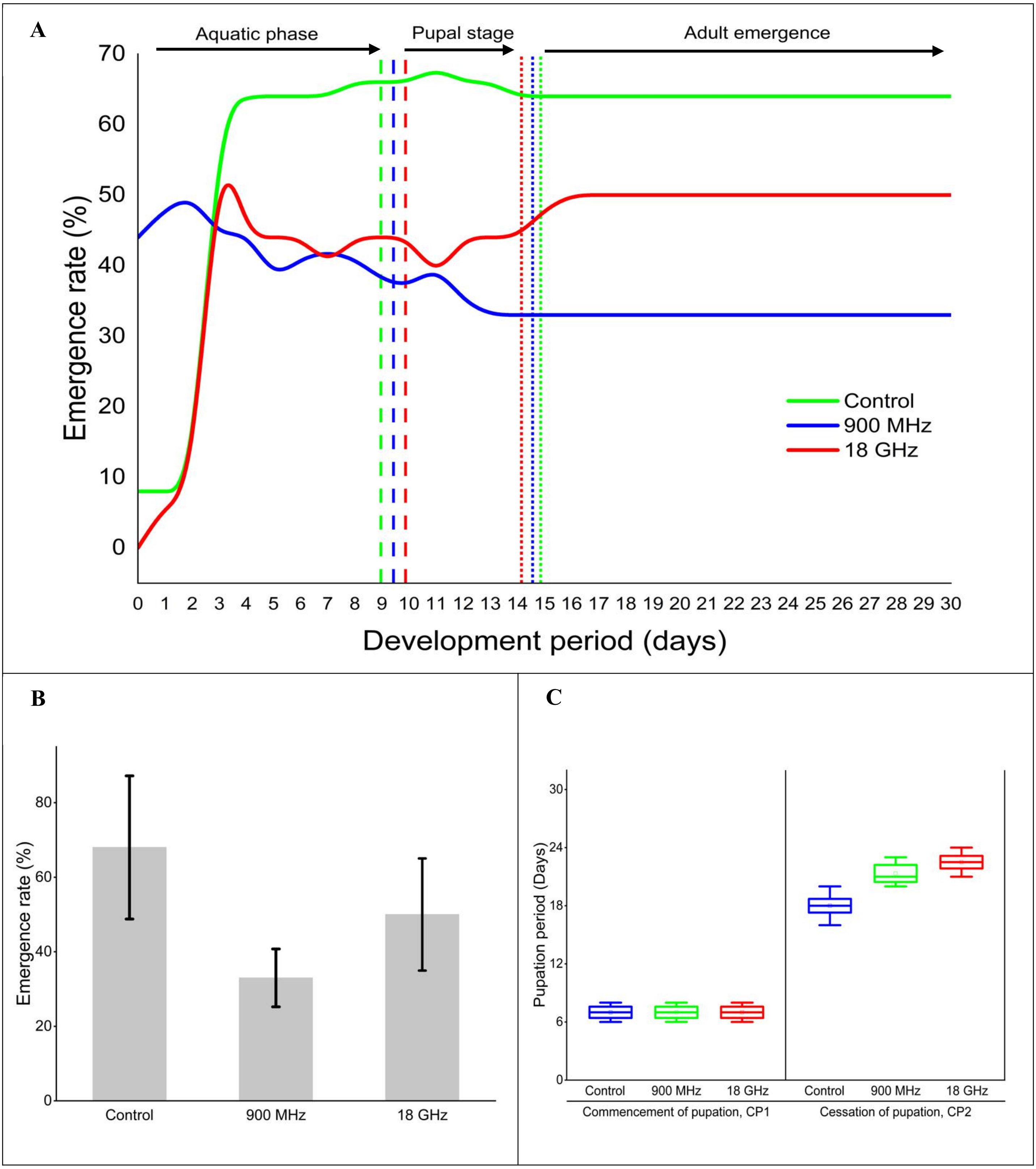

Supplement: Attachment — Submitted filename: PONE-D-23-36469.pdf [file pone.0298738.s001.pdf]
