# Supplementary material for: The impact of radiofrequency exposure on Aedes aegypti (Diptera: Culicidae) development
Source: PLoS One. 2024 Feb 27;19(2):e0298738. doi: 10.1371/journal.pone.0298738 (PMC10898727; doi:10.1371/journal.pone.0298738)
Supplement: Attachment — Submitted filename: PONE-D-23-36469_comments.pdf [file pone.0298738.s002.pdf]

Review of manuscript # PONE-D-23-36469 entitled "**Impact of Varying Radiofrequency Exposure on Aedes aegypti (Diptera: Culicidae) Development.**"

General comments:

1. The author should be including more details about nutrients used for larval development, how were they supplied? weight? volume? blood meal with mice, clarify if the expected time was to allow mating, ensure insemination of the females, time for oogenesis.
   Clarify, if the replications of the experiment design were at the same time, at different times on the same day, or on different days. How often was the water changed?
   How did you ensure that the sample size (unhatched eggs or larvae) was maintained after this manipulation?
2. The authors provide a comprehensive overview of the data analysis.
3. The manuscript presents grammar errors, and mistakes in usage of words, it is recommended to check the grammar throughout the document.
4. It is suggested to make the introduction completer and more concise, "what are the most important *Aedes* species worldwide? Transmitter dengue and other arboviruses, which ones? What are the traditional and innovative control methods?"
5. Did exposure to 18 GHz show the longest development time until pupation? How do the authors explain this regarding what they conclude?
6. The authors conclude "Our research has revealed that higher doses of RF radiation can expedite the hatching process of *Aedes* mosquitoes, while concurrently slowing down their overall developmental rate", what does it refer to? Larval development time? Life cycle? The results indicate larval development times between 9-10 days, and until the adult emergence between 14-15 days. This statement is contradictory.
7. closed laboratory populations (laboratory strain) are more conserved and adapted to controlled and favorable conditions, so their performance is usually higher. The authors should justify a little more the findings regarding the parameters of size and weight of the eggs since they could be directly influenced by the line of mosquitoes, their adaptations, and the diet of the females.
8. Another important consideration about the variability of hatching percentages is that they could be influenced by their storage time, given that as the days go by, they can lose viability, they can collapse, and the embryo can die. Hatching was slower, but it was not less than the control group. The reduced % hatching may not be due to RF, but rather to the aforementioned factors.
9. The authors point out that there is a positive correlation between lower exposure to RF, indicating shorter pupation time and adult emergence; however, it is contradictory when observing a longer pupation time at 38 GHz compared to 900 MHz, but not for emergence, in which control had the highest %, followed by 18GHz and 900 MHz
