# Supplementary material for: The impact of radiofrequency exposure on Aedes aegypti (Diptera: Culicidae) development
Source: PLoS One. 2024 Feb 27;19(2):e0298738. doi: 10.1371/journal.pone.0298738 (PMC10898727; doi:10.1371/journal.pone.0298738)
Supplement: Attachment — Submitted filename: Response to Reviewers.pdf [file pone.0298738.s003.pdf]

FAKULTI PERUBATAN DAN SAINS KESIHATAN

FACULTY OF MEDICINE AND HEALTH SCIENCES

PLOS ONE

Response to Reviewers 16 January 2024

I would like to submit the revised manuscript entitled "**The Impact of Radiofrequency Exposure on Aedes aegypti (Diptera: Culicidae) Development**" by Rahmat Dapari to be considered for publication as "an original article" in the PLOS ONE.

Below are the list of changes and rebuttal directed towards each point raised by the reviewer

Reviewer 1

No	Reviewer comments	Action taken
A1a	The author should be including more details about nutrients used for larval development, how were they supplied? weight? volume? blood meal with mice, clarify if the expected time was to allow mating, ensure insemination of the females, time for oogenesis.	All stages of larvae were subsequently fed daily until pupation, with an increasing feeding regime for each stage per day consisting of 0.06 to 0.12 mg per larva in L1-L2, 0.24 mg in L3, and 0.48 mg in L4. After a 72-hour mating period, a blood meal was administered by introducing a confined white mouse into the mosquito cage for 12 hours to ensure that the female mosquitoes were sexually mature and had laid eggs.
A1b	Clarify, if the replications of the experiment design were at the same time, at different times on the same day, or on different days. How often was the water changed?	The replications were at the same time on the same day. The pans were checked daily at the same hour of the day. Debris was removed using plastic pipettes, and water lost through evaporation was replaced daily to maintain constant levels
A1c	How did you ensure that the sample size (unhatched eggs or larvae) was maintained after this manipulation?	Standardized blood feeding (duration), control mating, and ensuring optimal environmental conditions of laboratory
A2	The authors provide a comprehensive overview of the data analysis.	The comment given by reviewer are taken
A3	The manuscript presents grammar errors, and mistakes in usage of words, it is recommended to check the grammar throughout the document.	The comment given by reviewer are taken and already corrected as suggestion.
A4	It is suggested to make the introduction completer and more concise, "what are the most important Aedes species worldwide? Transmitter dengue and other arboviruses, which ones?<br><br>What are the traditional and innovative control methods?"	*A. aegypti*, commonly known as the 'yellow fever mosquito' [3], plays a pivotal role in transmitting dengue fever while *A. albopictus* also known 'Asian tiger mosquito' is a secondary vector for these diseases.<br><br>To effectively manage *Aedes* mosquito populations traditional methods include source reduction, larvicides and biological control were applied. Recently, innovative approaches involve genetic control using Wolbachia bacteria to hinder virus transmission, employing spatial repellents, and utilizing GIS and remote sensing for larval source management. All these methods for a comprehensive approach to mosquito control.

A5	Did exposure to 18 GHz show the longest development time until pupation? How do the authors explain this regarding what they conclude?	The boxplot underscores that *A. aegypti* exposed to 18 GHz radiation did indeed show the longest development time until pupation.
A6	The authors conclude "Our research has revealed that higher doses of RF radiation can expedite the hatching process of Aedes mosquitoes, while concurrently slowing down their overall developmental rate", what does it refer to? Larval development time? Life cycle? The results indicate larval development times between 9-10 days, and until the adult emergence between 14-15 days. This statement is contradictory.	The sentence had been changed with this statement. Our research has revealed that 900 MHz RF exposure enhancing the hatching process of *A. aegypti* mosquitoes, while concurrently expedite the percentage of adult emergence.
A7	Closed laboratory populations (laboratory strain) are more conserved and adapted to controlled and favourable conditions, so their performance is usually higher. The authors should justify a little more the findings regarding the parameters of size and weight of the eggs since they could be directly influenced by the line of mosquitoes, their adaptations, and the diet of the females.	It was discovered that the laboratory strains of A. aegypti had more egg weight than the field strain, raising intriguing issues about the underlying processes determining mosquito reproductive features. This variation could be explained by a mix of environmental influences, genetic adaptation, and artificial selection. Mosquitoes experience stable conditions, abundant food supplies, and controlled temperature and humidity levels in a controlled laboratory setting, encouraging optimal nutrition of the females and resource availability for egg development. In the laboratory strain, this favourable environment may result in heavier eggs
A8	Another important consideration about the variability of hatching percentages is that they could be influenced by their storage time, given that as the days go by, they can lose viability, they can collapse, and the embryo can die. Hatching was slower, but it was not less than the control group. The reduced % hatching may not be due to RF, but rather to the aforementioned factors.	The variability in hatching percentages observed in mosquito eggs could be attributed to the duration of their storage. Over time, the viability of stored eggs may diminish, leading to a decrease in hatching success. This reduction could be a result of the eggs collapsing or the embryo perishing as storage time extends [28]. While hatching rates were observed to be slower, they did not fall below those of the control group. Therefore, it's plausible that the lower hatching percentages are not necessarily a consequence of radiofrequency (RF) exposure, but rather due to the factors related to prolonged storage and the associated decline in egg viability.
A9	The authors point out that there is a positive correlation between lower exposure to RF, indicating shorter pupation time and adult emergence; however, it is contradictory when observing a longer pupation time at 18 GHz compared to 900 MHz, but not for emergence, in which control had the highest %, followed by 18GHz and 900 MHz	Concerning the completion of pupation (CP2), the control group exhibited the shortest duration at 18 ± 1.58 days, followed by the 900 MHz group at 21.33 ± 1.53 days and the 18 GHz group at 22.5 ± 1.29 days.<br><br>Notably, the group exposed to 18 GHz completed the emergence of adult *A. aegypti* at 14.23 ± 1.04 days, whereas the 900 MHz group and the control group reached this stage at 14.56 ± 0.97 days and 14.78 ± 2.66 days, respectively.<br><br>Fig. 3B reveals the adultization rate, the control group had the higher adult emergence rate, at 68 ±1.33%. The 18 GHz exposure group revealed a lower adult emergence rate of 50 ±9.61% when compared to the control group. Furthermore, the adult emergence rate was 33 ±2.77% lowest in the group exposed to 900 MHz RF level

*Note: Any editing or changing in the main document that had been suggested by the first reviewer will be highlighted in blue colour.

Reviewer 2 (B)

No	Reviewer comments	Action taken
B1.	The paper is informative, generally well written. However, it can be greatly improved by improving statistical analyses. Without deeper tests, it would be very risky to make some conclusions like the existence of an effect of RF on eggs weight or the differences between wild and laboratory strains.	The comment given by reviewer are taken and already corrected as suggestion.
B2.	In the summary, you may consider rephrasing the last sentence for clarity:<br>Original: "Insects closely associated with humans, like mosquitoes, may experience increased RF absorption and dielectric heating."<br>Suggestion: "Insects closely associated with humans, such as mosquitoes, may experience increased RF absorption and dielectric heating."	The comment given by reviewer are taken and already corrected as suggestion.
B3.	Ae. Aegypti □ A. aegypti	The comment given by reviewer are taken and already corrected as suggestion.
B4.	Keywords: Eliminate RF as it's difficult to understand what it refers to.	The comment given by reviewer are taken and already corrected as suggestion.
B5.	"The use of a completely randomized design ensured that treatments were assigned to experimental units in a random and unbiased manner, enhancing the reliability and validity of the results. This design facilitated a systematic and controlled examination of how exposure duration and population variations impact the Ae. aegypti population, ultimately enhancing the study's comprehensiveness and reliability.''<br>This section could be deleted as it doesn't provide substantial information.	The comment given by reviewer are taken and already corrected as suggestion.
B6.	P18 and P19: Are the difference between egg weight of Ae. aegypti statistically significant or?	For instance, in the control group, the egg weight of the laboratory strain measured 12.40 µg ± 0.61, whereas the field strain recorded 11.97 ± 1.43 µg and the difference was statistically significant with p = 0.04. Similarly, in the 18 GHz RF exposure group, the weight of egg from the laboratory strain was 12.23 ±1.90 µg while for the field strain was 11.33 ±0.65 µg and the difference were statistically significant with p = 0.02. In contrast, in the groups exposed to 900 MHz RF, the laboratory strain egg with 12.60 ±0.40 µg was heavier than the field strain egg with 11.37 ±0.56 µg, but the difference was not statistically significant with p = 0.15
B7.	P22: The interval between the first and last hatching is too pong which makes the assessment of the effect of RF on hatching rate irrelevant. To overcome this situation, it's recommended to find a way to synchronize the development stage of different batches of eggs.<br>On possibility is to reduce the time between eggs harvests. In addition, the statistical analysis should be more advanced in the text and in the graphs.	The F2 eggs were used in the experimental procedures after a storage time between 2 and 3 weeks. The age of egg s batches used in this experiment were suspected to influence the hatching response. Because it was not possible to use eggs of uniform ae throughout the experiment, the age was included as an exclusion criterion<br>The F2 generation eggs of the *A. aegypti* strain were randomly allocated to the experimental group to ensure that the age of the eggs would not influence the outcomes
B8	P24: It's not clear if the difference in the weight is due to the strain (wild/laboratory) or to the radiation.	Specifically, when comparing the varied exposures for the laboratory and field strains, no statistically significant difference in egg length was identified between the control group and the groups exposed to 900 MHz or 18 GHz RF (p > 0.05). These findings

strongly suggest that the egg weight for both laboratory and field strains were not affected by RF exposure.

*Note: Any editing or changing in the main document that had been suggested by the second reviewer will be highlighted in orange colour.

Sincerely,

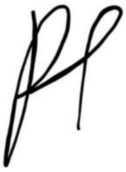

Dr. Rahmat Dapari
Principal Investigator,
Senior Medical Lecturer and Public Health Medicine Specialist
Department of Community health
Faculty of Medicine and Health Sciences
Universiti Putra Malaysia
+60192322815
drrahmat@upm.edu.my
